# What is the role of nephrologists and nurses of the dialysis department in providing fertility care to CKD patients? A questionnaire study among care providers

**DOI:** 10.1007/s11255-017-1577-z

**Published:** 2017-03-29

**Authors:** Gaby F. van Ek, Esmée M. Krouwel, Melianthe P. J. Nicolai, Brenda L. Den Oudsten, Marjolein E. M. Den Ouden, Sandra W. M. Dieben, Hein Putter, Rob C. M. Pelger, Henk W. Elzevier

**Affiliations:** 1 Department of Urology, Leiden University Medical Center, Albinusdreef 2, PO Box 9600, 2300 RC Leiden, The Netherlands; 2 Department of Medical and Clinical Psychology and Centre of Research on Psychological and Somatic Disorders, Tilburg University, Tilburg, The Netherlands; 3 Saxion, University of Applied Sciences, Enschede, The Netherlands; 4 Department of Gynecology, Leiden University Medical Center, Leiden, The Netherlands; 5 Department of Medical Statistics, Leiden University Medical Center, Leiden, The Netherlands

**Keywords:** Chronic kidney disease, Fertility care, Practice patterns, Questionnaires, Renal care providers

## Abstract

**Purpose:**

This study evaluated current fertility care for CKD patients by assessing the perspectives of nephrologists and nurses in the dialysis department.

**Methods:**

Two different surveys were distributed for this cross-sectional study among Dutch nephrologists (*N* = 312) and dialysis nurses (*N* = 1211).

**Results:**

Response rates were 50.9% (nephrologists) and 45.4% (nurses). Guidelines on fertility care were present in the departments of 9.0% of the nephrologists and 15.6% of the nurses. 61.7% of the nephrologists and 23.6% of the nurses informed ≥50% of their patients on potential changes in fertility due to a decline in renal function. Fertility subjects discussed by nephrologists included “wish to have children” (91.2%), “risk of pregnancy for patients’ health” (85.8%), and “inheritance of the disease” (81.4%). Barriers withholding nurses from discussing FD were based on “the age of the patient” (62.6%), “insufficient training” (55.2%), and “language and ethnicity” (51.6%). 29.2% of the nurses felt competent in discussing fertility, 8.3% had sufficient knowledge about fertility, and 75.7% needed to expand their knowledge. More knowledge and competence were associated with providing fertility health care (*p* < 0.01).

**Conclusions:**

In most nephrology departments, the guidelines to appoint which care provider should provide fertility care to CKD patients are absent. Fertility counseling is routinely provided by most nephrologists, nurses often skip this part of care mainly due to insufficiencies in self-imposed competence and knowledge and barriers based on cultural diversity. The outcomes identified a need for fertility guidelines in the nephrology department and training and education for nurses on providing fertility care.

## Introduction

Chronic kidney disease (CKD) is associated with a decrease in reproductive function [1–4]. Although fertility disorders (FD) are common in both male and female patients, the precise etiology is largely unknown. A major component is a disturbance in the hypothalamic–pituitary axis caused by CKD, resulting in menstrual irregularities, anovulation, and infertility in female patients [1]. For example, over 94% of female patients receiving dialysis experience menstrual irregularities [5]. If pregnancy does occur, patients are highly at risk for further deterioration of their renal function, pre-eclampsia, and the need for blood transfusions [1, 6, 7]. In addition, pregnancy and possible transfusions may complicate future renal transplantation as immunological sensitization might be induced [1]. The fetus is also at risk for intrauterine growth restrictions, along with multiple complications associated with preterm delivery [1, 6, 7]. In male CKD patients, besides disturbances in the hypothalamic–pituitary axis, oxidative stress and uremia both contribute to FD by decreasing testicular volume and impairment of spermatogenesis [2, 3].

Where little is documented on fertility management and counseling in male CKD patients, several studies exist on the management of fertility in female CKD patients [1, 8]. Counseling prior to conception is necessary for all female patients of reproductive age due to the complexity of fertility in CKD [1, 8]. Every disease stage requires different counseling, and adequate information on fertility is especially needed for those patients receiving renal replacement therapy [6]. Although FD are common during dialysis, prevention of unwanted pregnancy is necessary since recent innovations in dialysis induce hormonal normalization and thereby increase pregnancy rates up to 75% [1, 6, 7]. If pregnancy does occurs, early confirmation is of great importance as close monitoring of renal function, patients’ health, and the pregnancy itself is essential [1, 6, 7, 9].

Adequate fertility care, including counseling, management, and referral to a physician specialized in fertility, determines whether the pregnancy and maternal and fetal outcomes are successful [1]. A multidisciplinary approach is fundamental for success in this part of renal health care [1, 8]. Unfortunately, little is known about current fertility care for CKD patients provided by renal care providers as this subject is often ignored in research. No information is available on the current format of this part of renal care or the availability of guidelines appointing which care providers are accountable for addressing fertility issues. Since the nephrologist is a major renal care provider throughout all stages of CKD, it is most likely that the provision of fertility care depends on this renal care provider. However, considering the nephrologist’s increasing workload and a limited time available during consultation, nurses might play an important role in the future for providing fertility care for CKD patients. Nurses working in the dialysis unit could especially contribute to this part of renal care since they often have frequent and intensive one-on-one contact with their patients.

The aim of this multidisciplinary study was to examine current fertility care for CKD patients from the perspectives of the nephrologists and nurses working in the dialysis unit. Guidelines appointing who should provide fertility care within nephrology departments were focused on, as were practice patterns of nephrologists and nurses regarding informing, discussing, counseling on fertility, and referral to a physician specialized in fertility. In addition, the evaluation among nurses addressed possible barriers toward discussing fertility care and tools needed to provide it. Nurses’ competence, knowledge, and education regarding fertility in CKD patients were evaluated, as well as how these factors influence fertility care currently provided by them.

## Methods

### Study design

Data for this cross-sectional study were collected among nephrologists and nurses using two separate surveys. All practicing Dutch nephrologists (*n* = 318) who were member of the Dutch Federation of Nephrology (Nefrovisie) were requested to participate in the survey. A total of 312 questionnaires were sent to nephrologists’ home addresses; six obtained addresses were out of date. Non-respondents received a reminder letter two and/or four months after the initial mailing.

For the survey among nurses, all Dutch dialysis centers (both in and outside of the hospital) were approached for participation (*n* = 63). Thirty-four centers (54.0%) agreed on participation, and all their employed dialysis nurses and nurses specialized in nephrology received a questionnaire (*n* = 1171) at their work address. An e-mail was sent to non-responding centers received 2 and/or 4 months after the initial invitation as a reminder. Participating centers received a motivational e-mail after 4 months asking them to motivate their staff on returning the questionnaire. In addition, 40 questionnaires were handed out during an informative meeting for nurses specialized in nephrology resulting in a total of 1211 distributed questionnaires.

### Instrument design

The authors developed both questionnaires as validated instruments for assessing the aims of the studies were not available. The questionnaires were derived from instruments used in previous studies evaluating healthcare in medical departments [10–13]. Contents were based on issues described in the literature and additional topics identified by the authors. The classification of renal failure was according to K/DOQI clinical guidelines [9]. Both questionnaires were part of surveys that evaluated the discussion of sexual dysfunction as well. The items on sexual dysfunction were studied separately [14]. The questionnaire used in the nephrologist study was pilot-tested for content, length, and comprehensiveness by nephrologists and nephrology residents from the Leiden University Medical Centre (*n* = 7). No modifications were made as no remarks were made. The first sheet of the questionnaire contained demographic questions and an opt-out possibility. The (remaining) ten items assessed:

- Guidelines on fertility care within the nephrology department.
- Nephrologists’ practice patterns regarding informing, discussing, and counseling on fertility, including referral to physicians specialized in fertility.
- Fertility subjects addressed by the nephrologist.

The nurses’ questionnaire was pilot-tested at the Leiden University Medical Centre as well. Twenty-three nurses working in the dialysis department inspected layout, linguistics, and comprehensiveness of items. No comments were made, so the final questionnaire was identical. Demographic questions and an opt-out possibility were placed on the first sheet; the other ten questions assessed:

- Guidelines on fertility care within the dialysis or nephrology department.
- Nurses’ practice patterns regarding informing, discussing, and counseling on fertility with dialysis patients.
- Competence of the nurse in providing fertility care to dialysis patients.
- Possible barriers to discussing fertility with dialysis patients.
- Current knowledge and education on fertility in dialysis patients, including influence on fertility care currently provided by the nurse.
- Tools that could assist in providing fertility care to dialysis patients.

### Data analysis

Data were analyzed using IBM SPSS statistics 23 (SPSS Inc., Chicago, IL, USA). Descriptive statistics were used to describe demographic information and the answers given by the respondents. Bivariate associations were calculated using the Cochran–Armitage trend test. Two-sided *p* values of <0.05 were considered statistically significant.

### Ethical considerations

Since no patients or interventions were involved during both surveys, no formal ethical approval was needed in the Netherlands.

## Results

### Participation

In the nephrologist survey, a total of 159 of the 312 questionnaires sent were returned (response rate 50.9%). Thirty-eight respondents were not willing to participate, and reasons for not participating were, for instance, “lack of time” (*n* = 28), “not practicing” (*n* = 5), and “not interested” (*n* = 2). Fifteen respondents were excluded since they were specialized in pediatric nephrology. A total of 113 (36.2%) questionnaires were analyzed.

Of the 1211 questionnaires sent to the nurses, 550 were returned (response rate 45.4%). Twenty-three nurses declined participation, three questionnaires were excluded because less than 50% was filled in, and one respondent was excluded for not working as a nurse. Reasons not to participate were, for example, “no interest” (*n* = 7), “insufficient time” (*n* = 4), and “not enough experience” (*n* = 2). Including the 23 questionnaires from the pilot study, a total of 546 surveys were used for analyses. Demographic information of the respondents of both surveys is displayed in Table 1.

**Table 1:** Demographic characteristics

Nurses (*n* = 546)	*n* ^a^ (%)	Nephrologists (*n* = 113)	*n* ^a^ (%)
Gender		Gender	
Male	55 (10.1)	Male	70 (61.9)
Female	491 (89.9)	Female	43 (38.1)
Age (years)		Age (years)	
Median 47.0 (range 22–65)	542 (99.3)	Median 47.0 (range 33–62)	113 (100.0)
Mean 44.8		Mean 47.16	
Position		Position	
Dialysis nurse	491 (89.9)	Nephrologist	111 (98.2)
Nurse in dialysis registration training	16 (3.0)	Resident	2 (1.8)
Team leader dialysis department	17 (3.2)		
Nurse specialized in nephrology	19 (3.5)		
Other^b^	22 (3.9)		
Years of employment		Years of employment	
<1 year	13 (2.4)	<1 year	1 (0.9)
1–2 years	24 (4.4)	1–2 years	2 (1.8)
3–5 years	66 (12.1)	3–5 years	13 (11.5)
6–10 years	134 (24.5)	6–10 years	28 (24.8)
11–15 years	106 (19.4)	11–15 years	19 (16.8)
>15 years	203 (37.2)	>15 years	50 (44.2)
Clinical setting		Clinical setting	
University hospital	64 (11.7)	University hospital	27 (23.9)
District general teaching hospital	214 (39.2)	District general teaching hospital	54 (47.8)
District general hospital	184 (33.7)	District general hospital	30 (26.5)
Tertiary and district general hospital	6 (1.1)	Tertiary and district general hospital	1 (0.9)
Dialysis centre, outside the hospital	94 (17.2)	Dialysis centre, outside the hospital	6 (5.3)

^a^ *n* may differ due to multiple answers that could be given to questions or because the questions were not answered consistently, some were skipped or forgotten

^b^ Includes, e.g., diabetic nurse, pre-dialysis nurse, quality officer

### Guidelines on providing fertility care

Both surveys assessed if clear guidelines were present within nephrology departments regarding which care provider is accountable for discussing patients’ fertility. Ten nephrologists (9.0%) answered positively on the question if clear guidelines were present. Seven (6.3%) noted that their guidelines indicated the nephrologist was accountable for advising CKD patients on fertility matters, and one nephrologist (0.9%) noted the gynecologist was responsible. Of the nurses, 84 (15.6%) were aware of guidelines within their department regarding the accountability for discussing patients’ fertility. Thirty-one nurses (6.2%) stated these guidelines pointed out their own group of professionals as accountable. Other answers given concerning accountability were the “nephrologist” (*n* = 22, 4.4%), “nephrologist and nurse together” (*n* = 17, 3.4%), and “nephrologist, nurse and nephrology social worker” (*n* = 5, 1.0%).

### Providing information

Nephrologists and nurses were asked how often they informed their dialysis patients about the association between a decline in renal function, dialysis, and reduced fertility. Their answers on this question are listed in Table 2. More experienced nurses and nurses who were aware of guidelines regarding the accountability for discussing patients’ fertility informed their patients more often on reduced fertility (Linear-by-Linear Association, *p* = 0.004 resp. *p* < 0.001). No association was found between experience or awareness of guidelines and providing information among nephrologists.

**Table 2:** Providing information on fertility

How often do you inform your (dialysis) patients that a decline in renal function and dialysis are associated with reduced fertility?	Never *n* (%)	<50% of the cases *n* (%)	In 50% of the cases *n* (%)	>50% of the cases n (%)	Always *n* (%)
Nephrologists (*n* = 110)	12 (10.9)	30 (27.3)	16 (14.5)	27 (24.5)	25 (22.7)
Nurses (*n* = 537)	190 (35.3)	221 (41.0)	41 (7.6)	47 (8.7)	40 (7.4)

*n* differs because the questions were not answered consistently, and some were skipped or forgotten

### Discussion of fertility by the nephrologist

A majority of the nephrologists (*n* = 96, 88.1%) advised their female patients with renal failure not to become pregnant. Fifty-eight (62.4%) did so when their patients reached CKD stage 4 and 12.9% (*n* = 12) discouraged pregnancy in stage 5. Other nephrologists advised their patients to avoid pregnancy during stage 1 (*n* = 2, 2.2%), stage 2 (*n* = 1, 1.1%), and stage 3 (*n* = 20, 21.5%) of CKD. Besides advising their female patients to prevent pregnancy before renal transplantation, nephrologists discussed several other subjects regarding fertility with their female patients. These included: wish to have children (*n* = 103, 91.2%), health risks of pregnancy (*n* = 97, 85.8%), and inheritance of the disease (*n* = 92, 81.4%). With their male patients, they also discussed their wish to have children (*n* = 85, 75.2%) and inheritance of the disease (*n* = 76, 67.3%). On top of that, almost three-quarter of nephrologists (*n* = 84, 74.3%) inquired after erectile dysfunction. With regards to referring CKD patients to a physician specialized in fertility, on average the nephrologists referred 14.5% (SD 27.0) of female patients and 9.7% (SD 22.5) of male patients.

### Discussion of fertility by the nurse

Nurses were asked how often they discussed fertility with male and/or female patients and in how many cases the partner was present when they did. Answers are listed in Table 3.

**Table 3:** Discussion of fertility by the nurse

	Never *n* (%)	Seldom *n* (%)	Routinely *n* (%)	Often *n* (%)
Male patients	284 (53.2)	218 (40.8)	27 (5.1)	5 (0.9)
Female patients	235 (44.0)	253 (47.4)	37 (6.9)	9 (1.7)

	Never *n* (%)	<50% of the cases *n* (%)	≥50% of the cases^a^ *n* (%)	Always *n* (%)
How often is the partner present?	359 (72.2)	79 (15.9)	37 (7.4)	22 (4.4)

*n* differs because the questions were not answered consistently, and some were skipped or forgotten

^a^ ≥50% of the cases contains the answers “in 50% of the cases” and “more than 50% of the cases”

Nurses with more years of experience discussed fertility more often with male (Linear-by-Linear Association, *p* = 0.001) and female patients (Linear-by-Linear Association, *p* = 0.001). On the question “What could be helpful for the discussion of fertility”, 86.0% (*n* = 467) answered informative brochures, 55.6% (*n* = 302) thought training to discuss fertility would be helpful, and 15.1% (*n* = 82) recommended posters about fertility in the waiting room. Almost 45% (*n* = 242, 44.6%) felt that the nephrologist should initiate the conversation on fertility so nurses could then refer to that conversation. Self-reported answers to this question were, for example, “more privacy or private appointments to discuss fertility” (*n* = 22, 4.4%), “more knowledge” (*n* = 12, 2.4%), and “referral to a physician” (*n* = 4, 0.8%).

### Barriers experienced by the nurse

The survey contained a list of possible barriers that might prevent nurses from discussing fertility. They were asked to write down to which extent they agreed with the barriers. The barriers are listed in Table 4. Barriers most agreed on by the nurses were based on, “age of the patient” (62.6%), “insufficient training” (55.2%), “language and ethnicity” (51.9%), “insufficient knowledge” (49.4%) and “culture or religion” (47.1%).

**Table 4:** Barriers for nurses not to discuss fertility

	Agree *n* (%)^a^	Indecisive *n* (%)	Disagree *n* (%)^b^
Age of the patient	333 (62.6)	114 (21.4)	85 (16.0)
Insufficient training	295 (55.2)	146 (27.3)	93 (17.4)
Barriers based on language and ethnicity	275 (51.6)	178 (33.4)	80 (15.0)
Insufficient knowledge	265 (49.4)	164 (30.6)	107 (20.0)
Barriers based on culture or religion	251 (47.1)	175 (32.8)	107 (20.1)
Could not find a suitable moment	230 (43.4)	158 (29.8)	142 (26.8)
Patients do not bring up fertility spontaneously	219 (41.1)	170 (31.9)	144 (27.0)
Presence of a third person	209 (39.5)	148 (28.0)	172 (32.5)
Patient is too ill to discuss fertility	147 (27.5)	199 (37.3)	188 (35.2)
I feel uncomfortable to discuss fertility	135 (25.4)	170 (32.0)	227 (42.7)
No connection with the patient	124 (23.3)	151 (28.4)	257 (48.3)
Afraid to offend the patient	107 (20.1)	138 (25.9)	288 (54.0)
No referral options	106 (19.9)	143 (53.4)	285(53.4)
Patient is not ready to discuss fertility	104 (19.5)	244 (45.9)	184 (34.6)
Age difference between yourself and the patient	95 (17.8)	119 (22.3)	319 (59.8)
Someone else is accountable for discussing fertility	84 (15.8)	186 (34.9)	263 (49.3)
Changed fertility is not a problem for the patient	57 (10.7)	216 (40.6)	259 (48.7)
Insufficient time	50 (9.4)	117 (22.0)	366 (68.7)

*n* differs because the questions were not answered consistently, and some were skipped or forgotten

^a^ Agree contains the answers “totally agree” and “agree”

^b^ Disagree contains the answers “totally disagree” and “disagree”

### Knowledge and competence of the nurse

On the question “Is the subject fertility in CKD patients addressed during your training to become a nurse?” two-thirds of nurses (*n* = 351) answered affirmatively. Next, the nurses were asked to rate their own level of knowledge regarding fertility in CKD patients. Forty-seven nurses (8.7%) stated to have no knowledge at all, 254 (47.0%) answered “a little” and 194 (35.9%) had some knowledge. Forty-five nurses (8.3%) rated their own knowledge as sufficient and one nurse (0.2%) answered in having a lot of knowledge. Nearly 30% of the nurses (*n* = 156, 29.2%) felt competent to discuss fertility with their patients, three-quarters (*n* = 407, 75.7%) stated to be in need of expanding their knowledge. Nurses with less knowledge, who did not felt competent to discuss fertility or who were in need of expanding their knowledge, informed their patients less often on fertility. Furthermore, these nurses discussed fertility issues less often with their patients and noted “insufficient training” as a barrier to discuss fertility more often than other nurses. The *p* values ranged from <0.001 up to 0.01.

## Discussion

This analysis was the first to evaluate the extent of fertility care and information provided by nephrologists and nurses working in the dialysis unit for CKD patients. The study identified the absence of clear guidelines on this important part of renal health care in the majority of the Dutch nephrology and dialysis departments. Despite the lack of guidelines that appoint a health care professional to provide fertility care, the study shows that most nephrologists routinely discuss fertility during consultation. Unfortunately, this trend is not present throughout the whole nephrology department; nurses working in the dialysis unit who were unaware of guidelines or who had less work experience often skipped discussing fertility. However, the nurses are willing to provide this part of renal care in cooperation with the nephrologist. A suggestion was made that the nephrologists could initiate the subject of fertility enabling the nurses to continue the conversation during their own consultation with their patients. Still, multiple reasons exist that restrain nurses from assessing this subject. Besides the age of the patient, major barriers that withhold nurses from discussing fertility with their CKD patients derive from diversity in language, ethnicity, culture, and religion. Dealing with these factors in a diverse patient population requires certain skills, knowledge, and attitudes defined as cultural competence [15]. Cultural competence is becoming ever more important due to increasing cultural diversity among patients and care providers [15, 16]. This is especially so when providing fertility care, as sensitive issues need to be explained and discussed.

This study revealed the importance of training and nurses’ self-imposed knowledge and feelings of competence in providing fertility care. Unfortunately, study outcomes also indicated that these components were not self-evident among nurses of the dialysis department. This lack of training, self-imposed knowledge, and competence might be explained when focusing on the current educational system. Even though almost 70% of the nurses received education on fertility in CKD during their nurse training, a pressing need for additional training in discussing fertility exists. These lacuna in competence, knowledge and training regarding fertility issues is a problem not confined to the nurses working in nephrology; similar challenges arise among nurses in other medical departments as well [17, 18].

## Strengths and limitations

This study was a pioneer in assessing fertility care provided by renal care providers for CKD patients. In this light, a limitation of the study is the lack of formal comparison as no previous study of this nature has been conducted. Response bias may have occurred due to low responses in both surveys. However, this may also be interpreted as a lack of interest or knowledge regarding the subject. In addition, no validated questionnaires exist that assess the study aims. For this reason, two questionnaires were developed, which have not been formally validated. Validation of these two surveys was not performed as they will not be reused. Nevertheless, the authors attempted to develop reliable, literature-based and pilot-tested instruments. Finally, response bias may have occurred as socially desirable answers may have been given. As a result, answers might have been over or underestimated.

## Recommendations for practice

In order to achieve a cooperative fertility care system in CKD for both nephrologists and nurses, study results suggest several adjustments need to be made which involve both care providers. First, clear guidelines should be formulated within all nephrology departments to ensure multidisciplinary fertility care is provided. These guidelines should highlight the roles in providing fertility care of both nephrologists and nurses of the dialysis department, so any uncertainties regarding for instance accountability will be diminished. At the request of nurses themselves, informative brochures on fertility should be made available within nephrology departments and dialysis units to hand out to patients. In order to establish a functional system, nurses’ competence and knowledge regarding discussing fertility, especially in culturally diverse populations, should be improved. Renal care providers’ competence and skills regarding discussing fertility are of great importance as it is their ethical responsibility to explain the complexity of fertility in CKD to their patients [19]. Literature suggests that improvement of competence in discussing fertility and providing fertility care in culturally diverse populations could be achieved by providing adequate training; a recommendation confirmed by more than half of the nurses in this survey [15, 16, 20]. Discretion is called when providing fertility counseling as some patients could find being advised not to become pregnant by their physician as traumatizing [8, 21]. The lack of privacy is experienced by some of the nurses; this could call for the facilitation of a private and scheduled consultation to discuss a delicate subject such as fertility. In addition, CKD patients might benefit from an adequate referral system to physicians specialized in fertility [21, 22]. Wiles et al. [22] showed that in the UK referral to specialized pre-pregnancy counseling renal clinics resulted in highly satisfied female CKD patients. Awareness should be raised among renal care providers on the importance of referral to physicians specialized in fertility as currently only a small percentage of the CKD patients are referred. Finally, to be able to enhance current fertility care according to patients’ wishes, more research should be performed to determine their needs and preferences regarding this part of renal healthcare. Especially among male CKD patients more research is needed, as little is known about the preferable format, type and timing of fertility care for men.

## Conclusion

Guidelines that appoint who should provide fertility care to CKD patients are absent in the majority of Dutch nephrology departments. Current fertility care is not provided in a cooperative manner: most of the nephrologists assessed fertility routinely during consultation; less experienced nurses working in dialysis units often skipped this part of renal care. Besides a patients’ age, a lack of competence, insufficient knowledge and training, unawareness of guidelines, and barriers based on cultural diversity are reasons for nurses to omit providing fertility care. To achieve a cooperative fertility care system in CKD for both nephrologists and nurses, results emphasize the need for guidelines on this part of renal care in Dutch nephrology and dialysis departments and the provision of training to improve of nurses’ competence regarding cultural diversity and fertility. Finally, more research should be performed to determine patients’ needs and preferences regarding this important part of renal healthcare, especially among male patients.
